# Paroxysmal Nocturnal Hemoglobinuria: Diagnostic Challenges in Pediatric Patient

**DOI:** 10.1155/2019/4930494

**Published:** 2019-06-09

**Authors:** Dharshana Krishnaprasadh, Inna Kaminecki, Anna Sechser Perl, Jonathan Teitelbaum

**Affiliations:** ^1^Department of Pediatrics, The Unterberg Children's Hospital at Monmouth Medical Center, Long Branch, NJ, USA; ^2^Department of Pediatric Hematology Oncology, Saint Peters University Hospital, New Brunswick, NJ, USA; ^3^Department of Pediatric Gastroenterology, The Unterberg Children's Hospital at Monmouth Medical Center, Long Branch, NJ, USA

## Abstract

Paroxysmal nocturnal hemoglobinuria (PNH) is a rare, life-threatening hematologic stem cell disorder characterized by hemoglobinuria, thrombosis, and tendency for bone marrow failure. The rare incidence of PNH in children, its nonspecific clinical presentation, and occasional absence of hemoglobinuria make the diagnosis challenging. We present a case of a 17-year-old boy who was hospitalized with a history of recurrent abdominal pain, fever, and dark-colored urine. Laboratory tests revealed anemia, thrombocytopenia, and elevated inflammatory markers. Urinalysis was positive for protein and red blood cells, too many to be counted. Complement studies were within normal limits. Abdominal computed tomography showed a segment of the small bowel with wall thickening and signs of possible microperforation. Exploratory laparotomy revealed necrosis of the small bowel, and histological evaluation was suggestive of an autoimmune process with small vessel vasculitis. Bone marrow biopsy showed hypocellular marrow with a decreased number of myeloid cells, normal number of megakaryocytes, and signs of erythroid hyperplasia. Flow cytometry detected deficiency of CD59 leading to the diagnosis of PNH. The patient was treated with eculizumab infusions resulting in significant improvement. This case highlights the need for high clinical suspicion for rare entities such as PNH in patients presenting without hemoglobinuria.

## 1. Introduction

PNH is a rare hematopoietic disorder that originates from an acquired genetic mutation in a multipotent stem cell. It is characterized by an increased sensitivity of erythrocytes, to the hemolytic action of complement. Lack of complement inhibitors CD55 and CD59 on the blood cell surface is responsible for the clinical manifestations of the disease [1]. It affects both men and women equally. Although the condition can manifest at any age, it is commonly diagnosed in adulthood, with pediatric cases accounting for only 5–10% of the reported cases [2].

Clinical manifestations of PNH are nonspecific and include fatigue, abdominal pain, chest pain, renal insufficiency, and venous and arterial thrombosis. Laboratory evaluation is significant for hemolytic anemia, hemoglobinuria, and signs of bone marrow failure. As the symptoms of PNH are intermittent and nonspecific, initial presentation may not yield the correct diagnosis and requires a high index of suspicion.

## 2. Case Presentation

A 17-year-old Caucasian boy presented with several months of abdominal pain, fever, and dark-colored urine. Three months prior to this admission, he was hospitalized with similar complaints of epigastric abdominal pain, associated with vomiting, and fever. His initial CBC did not reveal pancytopenia and was within normal limits with WBC of 8.8 × 10^9^/L, hemoglobin of 110 g/dL, and platelet count of 155 × 10^9^/L.

While laboratory studies indicated the presence of anemia and thrombocytopenia, urinalysis revealed too numerous to count red blood cells. Abdominal CT showed normal-appearing kidneys and thickening of the wall of the small bowel, cecum, and ascending colon. In the context of persistent pancytopenia, fatigue, gross hematuria, and abdominal pain, our initial differential diagnosis included acute glomerulonephritis. Initial anemia was attributed to ongoing blood losses. Thrombocytopenia was attributed to acute illness. Differential diagnosis also included inflammatory bowel disease with anemia of chronic disease, intestinal lymphoma, vasculitis, and leukemia. *Clostridium difficile* toxin was detected by PCR in his stool. The patient was diagnosed with infectious colitis and IgA nephropathy. Cystoscopy was not performed as bladder pathology was low on our differential diagnosis. He was treated with metronidazole and discharged. The patient's gross hematuria and abdominal pain resolved, but he continued to have fatigue, anemia, and thrombocytopenia.

During his second presentation, the patient complained of severe abdominal pain, fever, and reappearance of dark-colored urine. He was a muscular teenage boy, with weight in the 84th percentile, height in the 95th percentile, and BMI in 95th percentile. On physical examination, he appeared alert, oriented, and in moderate distress due to abdominal pain. His abdomen was nondistended, soft, with tenderness on palpation in the left lower quadrant. No hepatosplenomegaly or lymphadenopathy was noted on exam.

Laboratory results showed a white blood cell count of 3.9 × 10^9^/L, hemoglobin of 96 g/dL, platelet count of 109 × 10^9^/L, and reticulocyte count of 4.1% (reference range, 0.5–2.5%). Differential count included 59% neutrophils, 13% bands, 22% lymphocytes, and 6% monocytes. Mean corpuscular volume noted to be 79.8 fl/cells. Serum ferritin noted to be 124 ng/ml. The erythrocyte sedimentation rate (ESR) was 56 mm/hr. Inflammatory markers were elevated, and C-reactive protein was 196.8 mg/L. Patient did not appear to be jaundiced on exam; however, his total bilirubin was elevated at 1.8 mg/dL with a direct bilirubin of 0.8 mg/dL. His serum lactate dehydrogenase was elevated at 1225 IU/L. With 13 mg/dL of blood urea nitrogen and 0.91 mg/dL of creatinine, his renal functions were within normal limits. Urine protein to creatinine ratio was normal at 0.15. His total bilirubin was 1.8 mg/dL (30.7 *μ*mol/L), and conjugated bilirubin was 0.8 mg/dL. Antistreptolysin O was 378 IU/ml (reference range, 0–200 IU/ml) and complement component 3 (C3) was 120 mg/dL (reference range, 85–183 mg/dL). The direct Coombs test was negative. A repeat urinalysis showed urine of amber color and too numerous to count red blood cells. Urine dipstick following microscopic urinalysis was performed. Dipstick was positive for 4 + blood. Microscopic urinalysis was positive for too numerous to count red blood cells. Hemoglobin and myoglobin were not additionally sent, as microscopic examination confirmed that there were too numerous to count red blood cells.

**Figure 1:**
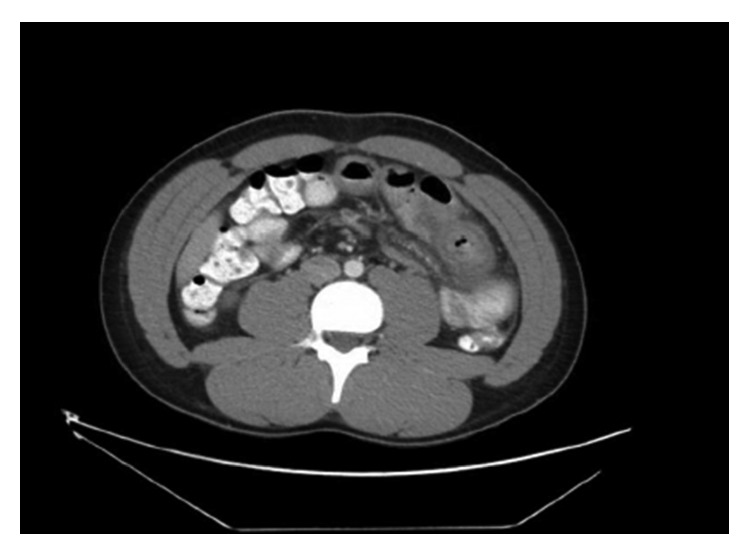
Abdominal computed tomography scan showing thickening of the small bowel wall.

**Figure 2:**
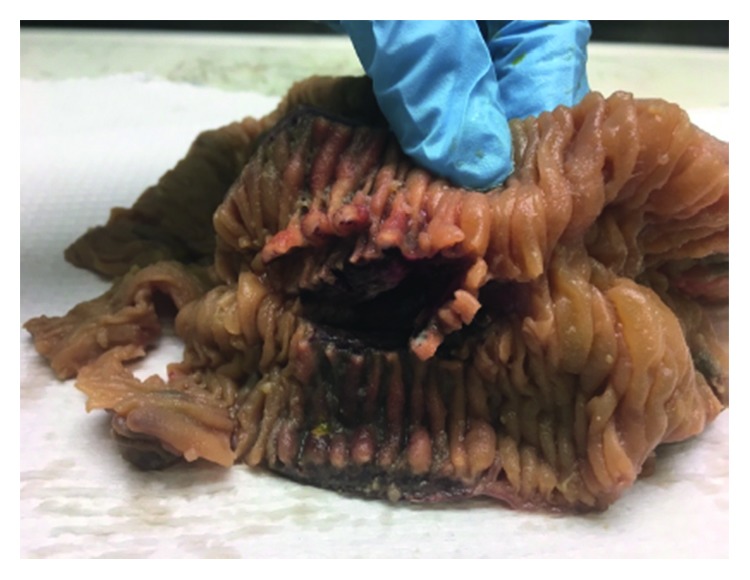
Necrotic segment of the small bowel.

**Figure 3:**
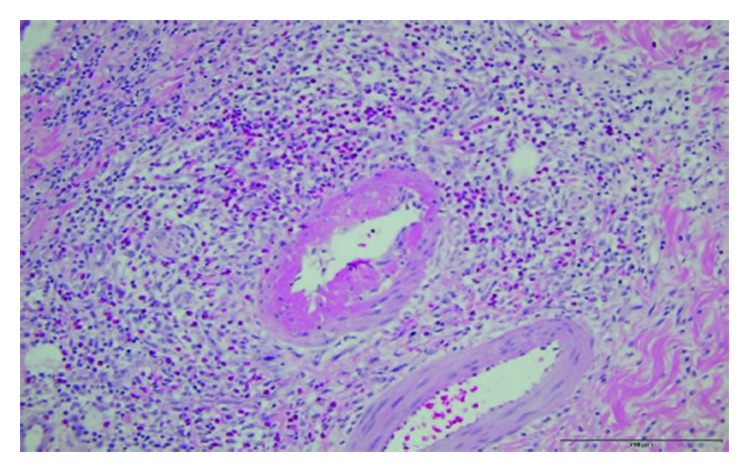
Histopathology slide showing hemorrhage, necrosis, and acute inflammation of the intestine.

Due to ongoing abdominal pain, a CT of the abdomen was performed, which revealed thickening of a segment of the small bowel wall and signs of possible microperforation (Figure 1). Due to worsening of abdominal pain, the onset of new peritoneal signs, and elevation of inflammatory markers, an exploratory laparotomy was performed. Surgical exploration showed a necrotic segment of the jejunum, 45 cm of the mid-jejunum was resected (Figure 2). Histopathology report confirmed the presence of hemorrhage, necrosis of the resected segment, and acute inflammation of the intestine and mesentery with the presence of eosinophils. Clinical presentation and histological evaluation were consistent with an autoimmune process with small vessel vasculitis (Figure 3). At this time, the differential diagnosis was broader and included polyarthritis nodosa, granulomatosis with polyangiitis, and eosinophilic granulomatosis with polyangiitis. After surgery, the patient continued to have pancytopenia and gross hematuria. Due to a further drop in hemoglobin (77 g/L), he required transfusion of packed red blood cells. To further determine the etiology of pancytopenia and hematuria, the hematology, gastroenterology, and nephrology services were contacted. The differential diagnosis was broad and included hematologic, rheumatologic, and neoplastic etiologies.

The antinuclear antibody titer was 1 : 80 (reference range, <1 : 40); anti-double-stranded DNA titer was 1 IU/ml (reference range, <4 IU/ml); haptoglobin level, <15 mg/dL (reference range, 30–200 mg/dL). The prothrombin time was mildly elevated at 16.9 seconds (reference range, 9.6–13.4 seconds), partial thromboplastin time was 23.7 seconds (reference range, 23.5–39.6 seconds), and the international normalized ratio was 1.5 (reference range, 0.9–1.1). Tests for hepatitis A, B, and C, Epstein-Barr, and human immunodeficiency viruses were all negative. Persistent pancytopenia in our patient triggered a bone marrow investigation. Bone marrow biopsy showed hypocellular marrow with a decreased number of myeloid cells, the normal number of megakaryocytes, and signs of erythroid hyperplasia. PNH flow cytometry panel results were pending, and the patient was discharged with the recommendation to follow-up with multiple subspecialists.

The patient later presented to an outside facility with recurrent abdominal pain and dark-colored urine. He also had generalized malaise and signs of upper respiratory symptoms, secondary to influenza infection, and CBC was remarkable for neutropenia with WBC 2,000 × 10^9^/L, with an absolute neutrophil count of 860/mcL . The urinalysis during his third hospital stay revealed hemoglobinuria. Although the urinalysis during the two previous hospitalizations was positive for too numerous to count red blood cells which was consistent with hematuria and not hemoglobinuria, the findings were consistent with a diagnosis of PNH: 22% GPI-deficient erythrocytes, 21.49% GPI-deficient granulocytes, and 50% GPI-deficient monocytes. Since patients with PNH have an interindividual (and in time) variable degree of bone marrow failure, they can also develop aplastic anemia. Our patient was noted to have an elevated reticulocyte count of 4.3%, which was attributed to his ongoing hemolysis. His elevated LDH levels may have been secondary to his hemolytic anemia and increased breakdown of red blood cells. The patient was started on eculizumab infusions for lifelong management. Repeat CH50 level was low at 8 Units, indicating the efficacy of eculizumab. During the first 2 months of treatment due to the high risk of thrombosis, he also received the anticoagulant enoxaparin. A repeat bone marrow evaluation showed normocellular marrow with normal trilineage hematopoiesis.

## 3. Discussion

PNH is a rare stem cell disorder that manifests as hemolytic anemia, thrombosis, and bone marrow failure. Hemolysis in PNH is complement-mediated and is a direct result of PNH cells acquiring a deficiency of complement regulatory proteins CD55 and CD59. The main roles of CD55 and CD59 proteins are to decrease the activity of complement C3 and to regulate the activity of the membrane attack complex, respectively. The absence of these two glycosylphosphatidylinositol-anchored proteins (GPI-APs) leads to uncontrolled complement activation that accounts for hemolysis and other PNH manifestations [1].

Almost all cases of PNH are the result of a somatic mutation in PIGA gene on the X chromosome [1], which accounts for the deficiency of complement inhibitory proteins CD55 and CD59. This leads to chronic complement-mediated hemolysis of the GPI-deficient erythrocytes, activation of platelets, monocytes, and granulocytes [1, 2]. The pathophysiology of the bone marrow failure in patients with PNH is not completely understood. Based on the leading hypothesis, PIGA mutation in the hematopoietic stem cells leads to the clonal dominance of PNH stem cells resulting in bone marrow failure [1]. Though the incidence of PNH in children and adolescents is rare, it is associated with higher morbidity and mortality [3, 4]. Ware et al. in a study involving the largest group of children and adolescents with PNH had reported that the mean age when children presented with PNH is 13 years [4]. Clinical presentation of PNH in children is different from that in adults. It has been reported that the prevalence of bone marrow failure is much higher in children and adolescents compared to adults with this disorder [4]. While 58% of children with PNH at presentation have bone marrow failure, only 25% of the adults have the same [4]. The reason for the increased prevalence of bone marrow failure in young patients with PNH is not completely understood at this time. The presence of peripheral pancytopenia and bone marrow hypoplasia can explain how PNH patients are initially diagnosed with aplastic anemia or myelodysplasia.

The classic symptoms of PNH are related to anemia, such as pallor, fatigue, and shortness of breath [1, 2, 5]. The percentage of erythrocytes lacking GPI-APs usually is between 2% and 100% [6]. Our patient had 22% complement-sensitive erythrocytes. As result of hemolysis, patients often present with hemoglobinuria, which is seen in 50% of the adults and only in 15% of children and adolescents [3, 4]. The absence of hemoglobinuria can attribute to delayed diagnosis of PNH in children. However, the presence of hemoglobinuria in patients with dark-colored urine also can be misdiagnosed with hematuria, often leading to a diagnosis of renal disease [6]. The mean delay in the diagnosis of PNH in children is reported to be 19 months since the initial presentation [4]. Our patient initially presented with dark-colored urine, and the urinalysis was positive for a large amount of blood and an uncountable number of red blood cells. Since most of the institutions do not run urine for hemoglobin/myoglobin routinely, it is prudent to order for these in a timely fashion. These findings were consistent with hematuria and initially led us to diagnose the condition as IgA nephropathy. Only 12 months later, the patient presented with dark-colored urine that was positive for hemoglobinuria. Our patient was diagnosed with PNH after 99 days of initial presentation, and this is very long for the diagnoses of complicated PNH. His initial presentation was unusual, as he had too numerous to count RBC on urine microscopy. Our index of suspicion for the diagnosis of PNH would have been higher on our list of differential diagnosis if he had had dark-appearing urine which was positive for blood on urine dipstick and negative for RBC on microscopy.

Thrombosis is the most common cause of mortality and morbidity in patients with PNH [7], and venous thrombosis is more common than arterial thrombosis (cerebral and coronary arteries). The risk of thrombosis appears to be lower in children than in adults [3]. Ware et al. have reported that only 31% of children and adolescents with PNH had confirmed venous thrombosis [4]. The mechanism of thrombosis in PNH is multifactorial and is not completely understood. Complement-mediated hemolysis, low nitric oxide levels, impairment of the fibrinolytic system, and inflammatory mediators are all responsible for the increased thrombotic risk in patients with PNH [5, 7]. While the most common site for thrombosis is the hepatic vein (Budd-Chiari syndrome), other common sites are the intraabdominal (portal, mesenteric, and splenic), cerebral (sagittal and cavernous sinus), and dermal veins. Depending on the site of thrombosis, patients may present with abdominal pain, splenomegaly, headache, and necrotic skin lesions. The risk of arterial thrombosis (cerebral and coronary arteries) is increased in patients with PNH but not as common as venous thrombosis. The major risk factor for thrombosis appears to be the size of the PNH clone and the degree of intravascular hemolysis [7]. Patients with more than 50% GPI-deficient granulocytes appear to be at the highest risk [5, 8], and absence of CD59 has been shown to be predominantly responsible for thrombosis in PNH [9]. During our patient's second hospitalization, the diagnostic laparotomy and pathological evaluation indicated mesenteric thrombosis. In our patient, we did not consider the diagnoses of PNH until after reviewing the small bowel resection pathology. In our literature review, we did not find information if complement inhibitor can reserve clots. Although he initially had only 21.49% of GPI-deficient granulocytes, the repeated panel for PNH showed 74% GPI-deficient granulocytes.

Another cause for PNH-associated abdominal pain can be smooth muscle dystonia, caused due to depletion of nitric oxide (NO) as a result of free hemoglobin release during episodes of hemolysis [7]. Low NO levels are also responsible for erectile dysfunction and pulmonary hypertension.

Other important clinical manifestations of PNH include infections. Infections can trigger activation of complement leading to episodic hemolysis. During the two initial hospitalizations, the patient presented with a sinus infection and was prescribed antibiotics. At the same time, he also had dark-colored urine, which retrospectively can be explained as caused by hemolysis followed by hemoglobinuria. It has been shown that 38% of children with PNH have serious infections including sinus, pulmonary, as well as systemic bacterial and fungal infections [4]. Associated complications from infections in patients with PNH can also be explained by neutropenia. As the patient presented with severe abdominal pain and radiological findings indicated a segment of the small bowel and colonic wall thickening, one of our differential diagnosis was infectious colitis. Others included Crohn's disease (which is associated with IgA nephropathy). Although the biopsy of the small intestine would be helpful, because jejunal lesion was not within reach of an endoscope, this would not be a safe procedure in the setting of a possible recent perforation . The stool antigen test was positive for *Clostridium difficile* toxin. Although the patient did not have diarrhea or blood in the stool, his clinical presentation was attributed to *Clostridium difficile* colitis. Interestingly, our review of the literature did not show any reported cases of PNH, positive for *Clostridium difficile*. It is possible that our patient developed *Clostridium difficile* colitis following the administration of antibiotics.

Laboratory findings in PNH include signs of hemolysis such as negative direct antiglobulin test, elevated levels of serum lactate dehydrogenase, elevated reticulocyte counts, low or absent serum haptoglobin, and hemoglobinuria [2]. Flow cytometry is the most sensitive and informative assay for diagnosis of PNH [10, 11]. PNH should be confirmed by peripheral blood flow cytometry to detect deficiency of CD55 and CD59 on >2 lineages. This test is usually performed by incubating the patient's blood cells with fluorescently labeled monoclonal antibodies that bind to GPI-APs proteins, which are typically missing in these patients. Detection of PNH clones in both the red blood cells and either the granulocyte or monocyte population is consistent with a diagnosis of PNH [5]. Usually there is a lag time between testing for PNH, and the results are typically available only after 7–10 days. Our patient was hemodynamically stable, and it was not justifiable to keep him hospitalized during this entire period.

Management of PNH depends on the severity of the symptoms. The most severe complication of PNH is thrombosis. Patients with symptoms of thrombosis should be promptly treated with anticoagulants. Patients with anemia may require red blood cell transfusions, as well as iron and folic acid supplementation. The main PNH treatment modality is anticomplement therapy. Eculizumab, a monoclonal antibody, is a terminal complement inhibitor and reduces intravascular hemolysis and risk of thrombosis [11, 12]. It inhibits the hemolytic effects of complement associated with CD59 deficiency. Our patient was treated with enoxaparin, an anticoagulant, along with eculizumab infusions. The efficacy of eculizumab was monitored by the complement CH50 assay. After initiation of management with eculizumab, the patient reported having more energy. He has not had any more episodes of dark-colored urine or abdominal pain. The level of CH50 was undetectable—8 Units during the second month after the eculizumab infusions, confirming the efficacy of the treatment. Although eculizumab is highly effective in the management of PNH, the only curative treatment for PNH is bone marrow transplantation [1].

## 4. Conclusion

PNH is a rare disorder, which may present with pancytopenia and a variety of clinical symptoms due to thrombosis. Occasionally, hemoglobinuria may be absent especially in the pediatric population, and therefore the initial presentation with PNH may not yield the diagnosis. This case highlights the need for high clinical suspicion for rarer entities presenting with uncommon manifestations. Patients with PNH will require lifelong treatment with eculizumab infusions followed by bone marrow transplantation.

## Supplementary Materials

Supplementary Materials Flowcytometric analysis results of the patient's bone marrow.

## References

[1] Brodsky R. A. (2014). Paroxysmal nocturnal hemoglobinuria. *Blood*.

[2] Parker C., Omine M., Richards S. (2005). Diagnosis and management of paroxysmal nocturnal hemoglobinuria. *Blood*.

[3] van den Heuvel-Eibrink M. M., Bredius R. G. M., te Winkel M. L. (2005). Childhood paroxysmal nocturnal haemoglobinuria (PNH), a report of 11 cases in the Netherlands. *British Journal of Haematology*.

[4] Ware R. E., Hall S. E., Rosse W. F. (1991). Paroxysmal nocturnal hemoglobinuria with onset in childhood and adolescence. *New England Journal of Medicine*.

[5] Brodsky R. A. (2008). Narrative review: paroxysmal nocturnal hemoglobinuria: the physiology of complement-related hemolytic anemia. *Annals of Internal Medicine*.

[6] Veerreddy P. (2013). Hemoglobinuria misidentified as hematuria: review of discolored urine and paroxysmal nocturnal hemoglobinuria. *Clinical Medicine Insights: Blood Disorders*.

[7] Hill A., Kelly R. J., Hillmen P. (2013). Thrombosis in paroxysmal nocturnal hemoglobinuria. *Blood*.

[8] Moyo V. M., Mukhina G. L., Garrett E. S., Brodsky R. A. (2004). Natural history of paroxysmal nocturnal haemoglobinuria using modern diagnostic assays. *British Journal of Haematology*.

[9] Hall S. E., Rosse W. F. (1996). The use of monoclonal antibodies and flow cytometry in the diagnosis of paroxysmal nocturnal hemoglobinuria. *Blood*.

[10] Richards S. J., Rawstron A. C., Hillmen P. (2000). Application of flow cytometry to the diagnosis of paroxysmal nocturnal hemoglobinuria. *Cytometry*.

[11] Young N. S., Meyers G., Schrezenmeier H., Hillmen P., Hill A. (2009). The management of paroxysmal nocturnal hemoglobinuria: recent advances in diagnosis and treatment and new hope for patients. *Seminars in Hematology*.

[12] Rother R. P., Rollins S. A., Mojcik C. F., Brodsky R. A., Bell L. (2007). Discovery and development of the complement inhibitor eculizumab for the treatment of paroxysmal nocturnal hemoglobinuria. *Nature Biotechnology*.

