# Supplementary material for: Paroxysmal Nocturnal Hemoglobinuria: Diagnostic Challenges in Pediatric Patient
Source: Case Rep Pediatr. 2019 Jun 9;2019:4930494. doi: 10.1155/2019/4930494 (PMC6590537; doi:10.1155/2019/4930494)
Supplement: Supplementary Materials — Flowcytometric analysis results of the patient's bone marrow. [file 4930494.f1.pdf]

Results

Flow cytometric analysis of the marrow reveals a small population (2.8% of total events) of CD45 dim to negative cells occupying the "blast gate." A subset of these cells (0.9% of total events representing 86 cells) co-express CD10 with CD20, consistent with mature B-cells and hematogones. Another small subset (0.5% of total events representing 50 cells) is positive for CD34 and CD33, consistent with myeloblasts.

The above results were obtained by using the following flow cytometry markers:
Tube #1: Kappa/Lambda, CD19, CD45
Tube #2: CD3, CD10 CD20, Cd45
Tube #3: CD2, CD5, CD7, CD45
Tube #4: CD13, CD33, CD34, CD45
Tube #5: HLA-DR, CD14, CD45, CD64
Tube #6: CD14, CD15, CD45, CD11b
INTRACELLULAR:
Tube #7: Isotypes
Tube #8: Tdt, CD3, CD45, CD79a
Tube #9: MPO, CD45

"This test was developed and its performance characteristics determined by (Monmouth Medical Center Laboratory). It has not been cleared or approved by the US FDA"

AS
RBC
09/20/16

Interpretation

NO FLOW CYTOMETRIC EVIDENCE OF ACUTE LEUKEMIA.
SEE ADDENDUM FOR PNH PANEL

INTERPRETATION:
CONSISTENT WITH PNH
